# Adolescence exposure to China’s great famine period and the association of metabolic syndrome in adulthood: a retrospective study

**DOI:** 10.1186/s12889-022-13047-6

**Published:** 2022-04-08

**Authors:** Ning Sun, Wei Li, Olatokunbo Osibogun, Mohammad Ebrahimi Kalan, Rime Jebai, Prem Gautam, Tanjila Taskin, Wupeng Yin, Jeffery A. Jones, Michelle Gamber, Wenjie Sun

**Affiliations:** 1 Department of Biostatistics, Robert Stempel College of Public Health, Florida International University, Miami, FL USA; 2 Department of Epidemiology, Robert Stempel College of Public Health, Florida International University, Miami, FL USA; 3 School of Medicine, Linberger Comprehensive Cancer Center, University of North Carolina in Chapel Hill, Chapel Hill, NC USA; 4 Department of Health Policy and Community Health, Jiann-Ping Hsu College of Public Health, Georgia Southern University, Statesboro, GA USA; 5 School of Health Professions, Shenandoah University, 1775 North Sector Court, Suite 220 B, Winchester, VA 22601 USA; 6 Center for Health Sciences, Oklahoma State University, 1111 W. 17th Street, Tulsa, OK 74017 USA

**Keywords:** Famine, Metabolic syndrome, Aging Chinese, Sex difference

## Abstract

**Background:**

Exposure to famine during early life is related to several adverse health outcomes in adulthood, but the effect of famine exposure during adolescence is unclear. This study aims to examine whether exposure to famine in adolescence is associated with metabolic syndrome (MetS) in adulthood.

**Methods:**

This study included 4130 Chinese adults (2059 males and 2071 females) aged 59–71 from the 2011 China Health and Retirement Longitudinal Study (CHARLS). All the selected participants were exposed to the three-year time period (1959–1961) of China’s Great Famine. Participants were categorized into an adolescent-exposed group (born 01/01/1944–12/31/1948) and a non-adolescent-exposed group (born 01/01/1940–12/31/1941 and 01/01/1951–12/31/1952). Sex-stratified multiple logistic regression models were used to estimate the association between exposure to famine in adolescence and MetS.

**Results:**

Participants exposed to famine during adolescence were more likely to report MetS (aOR = 1.35; 95%CI 1.01–1.78) compared to the non-adolescent-exposed group. Further, males were 45% less likely to report MetS than females (aOR = 0.55; 95%CI 0.36–0.83). After stratification by sex, the effects of famine exposure during adolescence on MetS were detected among males only (aOR = 1.97; 95%CI 1.20–3.24). Additionally, males with a history of drinking were more likely to report MetS compared to those with no history of drinking (aOR = 2.63; 95%CI 1.41–4.90).

**Conclusions:**

Our findings reveal that exposure to famine during adolescence is associated with higher odds of MetS in adulthood overall, and this association is only pronounced among males. This study emphasizes that undernutrition in early life, including adolescence, may have a long-term effect and be associated with adverse health events in middle-to-late life. Targeting those elderly people who suffered famine during adolescence may help prevent the development of MetS in later life.

## Background

Metabolic syndrome (MetS) comprises a group of disorders, including abdominal obesity, hypertension, dyslipidemia, and insulin resistance [1]. It is widely used to highlight the risk of patients developing cardiovascular disease (CVD) and type 2 diabetes (T2DM) all over the world [2]. According to a systematic review conducted in Asia–Pacific countries, the prevalence of MetS was approximately 21.3% in China [3] in 2009, ranging from 6.17% for Tibetan Chinese to 35.42% for Korean Chinese [4]. A considerable body of evidence shows a strong link between MetS and CVD development, T2DM, and other conditions such as nonalcoholic fatty liver disease and hypogonadism [5]. Therefore, to prevent the health conditions mentioned above and improve life expectancy during adulthood, it is pivotal to understand the factors associated with MetS over time.

China’s Great Famine was a period from 1959 to 1961 and caused millions of deaths [6]. In the last decades, accumulating studies have explored the effect of early-life exposure to famine on MetS in adulthood [7, 8]. However, findings on the effect of famine exposure are not consistent. For example, a study conducted among a Dutch famine cohort examined the effect of fetal famine exposure on MetS and observed no statistical significance [9]. In contrast, studies conducted in Chinese populations found that exposure to famine in early life (e.g., during fetal life) was associated with an increased risk of MetS in adulthood [7, 8]. To date, most of the studies have been focused on examining the effect of famine in early life (e.g., fetal and childhood period), and evidence in adolescents is limited. One study from a Korean cohort found that fetal and early childhood exposure increased the risk of MetS, but this association was not significant among the adolescent-exposed group [10]. Another study examined the relationship between famine exposure and MetS by combining the adolescence period with young adulthood and did not find statistical significance [11]. However, a study conducted in China observed a marginal effect on the risk of MetS among adolescents exposed to famine [12]. Adolescent exposure to famine is also related to abdominal obesity [13], diabetes [14], and dyslipidemia [15], which are the important components of MetS. Furthermore, the relationship between famine exposure and MetS also differs by sex. Prior research has shown that women exposed to famine during early life had a significantly higher prevalence of MetS [7, 12], while this association was not detected among men [7, 12].

The China Health and Retirement Longitudinal Study (CHARLS) has been broadly used to examine famine in different age groups and the associated adverse outcomes [16–19]. Building on previous literature, our study used data from CHARLS to 1) explore the relationship between famine exposure during adolescence and the association of MetS in adulthood measured by Chinese Diabetes Society (CDS) 2004 criteria [20], and 2) to assess sex-related differences in the relationship.

## Methods

### Study sample and the CHARLS survey

This study was a retrospective, cross-sectional study conducted using the baseline dataset from the CHARLS. The CHARLS is a national longitudinal survey of individuals older than 45 years of age and their spouses, including 10,000 households and 18,245 individuals in 150 counties/districts and 450 villages/resident committees [21]. The CHARLS survey supports the collection of scientific research on older persons in China. The baseline was collected in 2011, and participants were followed up every 2 years until 2018 (4 waves of data collection). Samples were chosen through multistage probability sampling and data were collected by face-to-face computer-assisted personal interviews (CAPI). The study protocol is performed in accordance with relevant national guidelines. Data used in this study has been anonymized and is publicly available. Further details about the CHARLS data are available elsewhere [21].

### Famine cohorts

We categorized famine exposure into two groups (an adolescent-exposed cohort vs. a non-adolescent-exposed cohort). The cohorts were divided according to the participants’ birth dates. According to privous literature [11, 12, 22–24], participants born between 01/01/1944 and 12/31/1948 were classified as the adolescent-exposed cohort, including ages between 11–17 years old through the entire famine period (1959–1961). Participants born between 01/01/1940 and 12/31/1941 and between 01/01/1951 and 12/31/1952 were combined as a non-adolescent-exposed cohort to balance the age difference between exposed and non-exposed groups [25]. We excluded the participants who were born in 1942–1943 and 1949–1950 because they did not experience the entire period of adolescence during the three-year famine time.

### Metabolic Syndrome (MetS)

Since the samples were collected in China, we used the Chinese Diabetes Society (CDS) criteria [20] of MetS: *Body Mass Index (BMI)* ≥ 25.0 kg/m^2^, *dyslipidemia*: fasting blood triglycerides (TG) ≥ 1.7 mmol/L and (or) fasting blood high-density lipoproteins cholesterol (HDL-C) < 0.9 mmol/L for male or < 1.0 mmol/L for female, or a self-reported doctors’ diagnosis of dyslipidemia; *high blood pressure*: systolic blood pressure ≥ 140 mmHg and/or diastolic pressure ≥ 90 mmHg, or a self-reported doctors’ diagnosis of hypertension; and *high blood glucose level*: fasting plasma glucose (FPG) ≥ 6.1 mmol/L and (or) 2 h PG ≥ 7.8 mmol/L, or a self-reported doctor’s diagnosis of diabetes [20]. Meeting ≥ 3 criteria was defined as MetS. In addition to BMI, data for the other three criteria were obtained from the following self-reported questions: “Have you ever been diagnosed with hypertension/dyslipidemia/diabetes by a doctor? (Yes/No)”.

### Covariates

Demographic characteristics included biological sex (male and female), education (less than elementary/elementary/middle school/higher than middle school), marital status (married and living with spouse/married but not living with spouse/separated/divorced/widowed/never married), and residence (rural and urban). Physical activity was categorized as insufficient, light, moderate, and vigorous levels according to previous literature from the CHARLS [26]. For example, at least 10 min of vigorous physical activity on any day of a usual week was considered vigorous level. Smoking status was classified as current smoker (smoked in past 30 days), former smoker, and never smoker. Drinking status was grouped as current low frequency (< 5 drinks per month), current high frequency (≥ 5 drinks per month), never, and quitter based on the Substance Abuse and Mental Health Services Administration (SAMHSA) [27].

### Statistical analyses

Statistical analyses were performed using SAS 9.4 (SAS Institute, Cary, NC, USA). Numbers and percentages were presented for the basic characteristics related to famine exposure. Chi-square tests were employed to detect the association between each variable and MetS. Sex-stratified multivariable logistic regression models were used to assess the association between famine exposure and MetS with adjustment of demographic variables and behavioral factors. Adjusted odds ratios (aORs) and corresponding 95% confidence intervals (95%Cis) were reported. Two-tailed *P* < 0.05 was set as statistically significant throughout the analyses.

## Results

Table 1 shows the characteristics of MetS and its components. Except for diabetes, the adolescent-exposed group had higher proportions of MetS, obesity, hypertension and dyslipidemia compared to the non-adolecent-exposed group.

**Table 1:** Characteristics of MetS and its components

		**Non-exposed group (*n* = 1,957)**	**Exposed group (*n* = 2,176)**	*p*-value*
MetS	No	1859 (94.99)	2041 (93.80)	0.096
	Yes	98 (5.01)	135 (6.20)	
Obesity	No	1519 (77.62)	1637 (75.23)	0.071
	Yes	438 (22.38)	539 (24.77)	
Hypertension	No	1388 (71.69)	1457 (67.67)	0.005
	Yes	548 (28.31)	696 (32.33)	
Diabetes	No	1780 (92.52)	1987 (92.63)	0.886
	Yes	144 (7.48)	158 (7.37)	
Dyslipidemia	No	1704 (89.36)	1862 (87.42)	0.056
	Yes	203 (10.64)	268 (12.58)	

^*^P-values are based on Chi-squared tests

Table 2 shows the characteristics of the study sample (*N* = 4,130). There were 2,059 males and 2,071 females in this study. For males, the prevalence of MetS was 5.42% and 3.26% for the adolescent-exposed group and the non-adolescent-exposed group, respectively. For females, the prevalence of MetS was 7.02% and 6.58%, respectively. Higher proportions were observed among females compared to males in both exposed (63.16% vs. 32.97%) and non-exposed groups (69.59% vs. 39.43%) with less than elementary education. Among males, both the exposed and non-exposed groups had a higher percentage of current smokers and drinkers compared to females.

**Table 2:** Basic characteristics related to famine exposure for the sex-stratified sample (*n* = 4,130)

	**Male (*N* = 2,059)**			**Female (*N* = 2,071)**		
	**Exposed group**^**a**^** (*n* = 1,108)**	**Non-exposed group**^**b**^ **(*n* = 951)**	***p*-value**	**Exposed group (*n* = 1,068)**	**Non-exposed group (*n* = 1,003)**	***p*-value**
	**n (%)**	**n (%)**		**n (%)**	**n (%)**	
**Metabolic Syndrome**						
Yes	60 (5.42)	31 (3.26)	0.018	75 (7.02)	66 (6.58)	0.690
No	1048 (94.58)	920 (96.74)		993 (92.98)	937 (93.42)	
**Education**						
Less elementary	365 (32.97)	375 (39.43)	< .001	672 (63.16)	698 (69.59)	< .001
Elementary	419 (37.85)	274 (28.81)		236 (22.18)	160 (15.95)	
Middle school	200 (18.07)	196 (20.61)		111 (10.43)	90 (8.97)	
Over high School	123 (11.11)	106 (11.15)		45 (4.23)	55 (5.48)	
**Marital Status**						
Married with spouse present	946 (85.38)	815 (85.70)	0.992	832 (78.05)	779 (77.74)	0.108
Married not living with spouse	60 (5.42)	52 (5.47)		38 (3.56)	50 (4.99)	
Separated/Divorced/Widowed	82 (7.40)	67 (7.05)		195 (18.29)	168 (16.77)	
Never	20 (1.81)	17 (1.79)		1 (0.09)	5 (0.50)	
**Residence**						
Rural	869 (78.43)	750 (79.11)	0.705	821 (76.94)	760 (75.85)	0.557
Urban	239 (21.57)	198 (20.89)		246 (23.06)	242 (24.15)	
**Smoking Status**						
Current	565 (53.81)	486 (53.82)	0.643	79 (7.50)	79 (7.95)	0.114
Quit/Former	201 (19.14)	160 (17.72)		20 (1.90)	33 (3.32)	
Never	284 (27.05)	257 (28.46)		955 (90.61)	882 (88.73)	
**Drinking Status**						
Current high frequency	257 (26.80)	205 (24.91)	< .001	33 (3.23)	24 (2.47)	0.490
Current low frequency	197 (20.54)	164 (19.93)		76 (7.44)	81 (8.32)	
Quit/Former	164 (17.10)	94 (11.42)		36 (3.52)	27 (2.77)	
Never	341 (35.56)	360 (43.74)		877 (85.81)	841 (86.43)	
**Physical Activity**						
Light	107 (9.66)	82 (8.62)	0.167	128 (11.99)	132 (13.16)	0.878
Moderate	131 (11.82)	92 (9.67)		147 (13.76)	134 (13.36)	
Vigorous	149 (13.45)	152 (15.98)		114 (10.67)	107 (10.67)	
Insufficient	721 (65.07)	625 (65.72)		679 (63.58)	630 (62.81)	

^a^Exposed group refers to adolescent-exposed group; ^b^Non-exposed group refers to non-adolescent-exposed group

Table 3 shows the results that were yielded from the ultivariable logistic regression models. Overall, participants exposed to famine during adolescence were 35% more likely to report MetS compared to those in the non-adolescent-exposed group (aOR = 1.35; 95%CI 1.01–1.78). Overall, males were 45% less likely to report MetS than females (aOR = 0.55; 95%CI 0.36–0.83). Participants who reported being separated from their spouse, divorced, or widowed (aOR = 0.50; 95%CI 0.30–0.82), living in a rural area (aOR = 0.60; 95%CI 0.43–0.84), and having vigorous physical activity (aOR = 0.50; 95%CI 0.28–0.92) were less likely to report MetS compared to the reference groups. Participants with elementary education (aOR = 1.45; 95%CI 1.02–2.06) and those who had quit drinking (aOR = 1.91; 95%CI 1.19–3.05) were more likely to report MetS.

**Table 3:** Multivariate logistic regression assessing the association between adolescent-exposed famine and MetS

Variables	Model A	Model B	Model C
	**Adjusted Odds Ratio (95%CI)**^**#**^	**Adjusted Odds Ratio (95%CI)**	**Adjusted Odds Ratio (95%CI)**
**Famine**			
Non-adolescent-exposed	Ref	Ref	Ref
Adolescent-exposed	1.35 (1.01–1.78)^*^	1.97 (1.20–3.24)**	1.08 (0.76–1.54)
**Biological sex**			
Female	Ref	-	-
Male	0.55 (0.36–0.83)**	**-**	-
**Marital Status**			
Married with spouse present	Ref	Ref	Ref
Never	0.65 (0.09–4.90)	0.82 (0.11–6.42)	NA
Married not living with spouse	0.59 (0.24–1.46)	0.39 (0.05–2.84)	0.70 (0.25–1.97)
Separated/Divorced/Widowed	0.50 (0.30–0.82)**	0.47 (0.14–1.54)	0.52 (0.30–0.90)*
**Education**			
< Elementary	Ref	Ref	Ref
Elementary	1.45 (1.02–2.06)*	1.05 (0.58–1.91)	1.70 (1.10–2.62)*
Middle school	1.41 (0.92–2.15)	1.21 (0.62–2.36)	1.50 (0.86–2.62)
> = High School	1.57 (0.93–2.64)	1.68 (0.79–3.55)	1.25 (0.58–2.70)
**Residence**			
Urban	Ref	Ref	Ref
Rural	0.60 (0.43–0.84)^**^	0.68 (0.39–1.18)	0.61 (0.40–0.92)*
**Physical Activity**			
Insufficient	Ref	Ref	Ref
Light	1.24 (0.84–1.83)	0.96 (0.46–2.03)	1.38 (0.87–2.19)
Moderate	1.08 (0.72–1.63)	1.22 (0.63–2.37)	1.02 (0.60–1.71)
Vigorous	0.50 (0.28–0.92)*	0.74 (0.34–1.60)	0.30 (0.11–0.84)*
**Drinking Status**			
Never	Ref	Ref	Ref
Current high frequency	1.12 (0.67–1.85)	1.60 (0.86–2.98)	0.25 (0.03–1.84)
Current low frequency	0.80 (0.49–1.33)	1.00 (0.48–2.07)	0.75 (0.35–1.57)
Quit	1.91 (1.19–3.05)**	2.63 (1.41–4.90)**	1.21 (0.47–3.13)
**Smoking Status**			
Never	Ref	Ref	Ref
Current	0.64 (0.41–1.01)	0.59 (0.34–1.05)	0.63 (0.27–1.46)
Quit	1.53 (0.95–2.46)	1.37 (0.76–2.46)	1.70 (0.70–4.17)

Bolded point estimates indicate statistical significance at *P* < 0.05; ^#^95%CI: 95% confidence interval; ^*^P < 0.05, ^**^P < 0.01

Model A: Adjusted for gender, education, marital status, smoking status, drinking status,residence, and physical activity

Model B: Adjusted for education, marital status, smoking status, drinking status,residence, and physical activity among males

Model C: Adjusted for education, marital status, smoking status, drinking status,residence, and physical activity among females

After stratification by sex, the effects of famine exposure during adolescence on MetS were detected among males only (aOR = 1.97; 95%CI 1.20–3.24). Former male drinkers were more likely to report MetS compared to those who did not report drinking (aOR = 2.63; 95%CI 1.41–4.90). All other covariates in the male subgroup were not significant. Among female participants, most results were consistent with the overall sample such as marital status, education, residence and physical activity. However, no significant association between exposure to famine during adolescence and MetS was detected among females (aOR = 1.08; 95%CI 0.76–1.54).

## Discussion

In this study, a significant association between famine exposure during adolescence and MetS was identified, with this association only consistent among males after stratification by sex. Males who spent their period of adolescence during the famine were 1.97 times more likely to report MetS in adulthood compared to the non-adolescent-exposed group. This highlights that the negative effects of famine may extend beyond the “first 1000 days” of life that is often the window of development considered the most critical and impactful to later health outcomes [28]. Similar to those participants with famine exposure in the fetal or early stage of childhood, the adolescent period could also affect survivors’ health during adulthood.

Although research indicates that being overweight or obese in early life is a predictor of MetS in adults [29], poor or inadequate nutrition in early life can also cause MetS in later life [30]. This highlights the potential role of biological mechanism during the famine such as an indicator of inadequate intake of energy. A previous study pointed out that participants with MetS showed a lower intake of energy, carbohydrates, and fiber than participants without MetS [31]. The adolescence period may be marked by a time when adolescents are in the process of establishing responsibility for their health, including decisions around diet [32]. Undernutrition in this period can lead to longstanding physiologic and psychologic stress and therefore changes the production of hormones such as thyroid hormones [32]. Established evidence shows that abnormal thyroid function could have an impact on the components of MetS through a variety of effects on energy homeostasis, lipid and glucose metabolism, blood pressure and plasma cholesterol levels [33, 34]. Therefore, undernutrition or nutrition deprivation at an early stage may contribute to the development of MetS in later life. A previous study suggested that monitoring the MetS components during thyroid dysfunction could help prevent MetS [34]. Providing adequate nutritional supplements in early stages of life such as during adolescence might profoundly reduce the risk of MetS in later life.

Previous studies have suggested boys may be more susceptible to shortages of energy, while girls are more sensitive to micronutrient deficiencies such as iodine and iron [35, 36]. Literature also reported the potential effects of famine on MetS in both sexes [12], while in this current study it was only observed among males. A possible explanation for the inconsistency between this study analysis of the CHARLS data and previous studies is this study’s focus on famine exposure during adolescence. Girls' development is faster than boys, so they may be more mature in adolescence [37, 38], and therefore they could be less likely to be affected by famine exposure. Additionally, consistent with the findings observed in previous studies [39, 40], the sex difference in MetS might be due to the discrepancies in the levels of hormones (e.g., sex hormone or growth hormone)-a vital role in the development of MetS [41, 42]. MetS, in essence, is a disorder in the metabolic system regulated by those hormones through negative or positive feedback, such as thyroid hormone [43]. This sex difference in MetS may be impacted by those hormones through direct or indirect regulation. Thyroid hormones regulate metabolism including metabolic rate, appetite control, and sympathetic activity [44]. Consequently, it may lead to adiposity, which is developed and secreted during puberty [44]. Another alternative explanation for the sex difference of MetS could be due to selenium or glutathione peroxidase [45]. Selenium is one of the key components of glutathione peroxidase and its balance is altered by sex [45]. Glutathione peroxidase appears to have a dual role in MetS, particularly in glucose and lipid metabolism [45]. While the evidence from animal studies is available, this hypothesis lacks evidence from human studies.

Education, physical activity, living areas, and marital status were found significantly associated with MetS only among females. Among males, we only observed one significant covariate: former drinkers were more likely to report MetS compared to those who did not drink alcohol. Former drinkers may engage in substitution behaviors that could increase the risk of MetS or some of its components, such as diabetes and hypertension [46]. Former drinkers may also have given up alcohol consumption after a MetS diagnosis to limit carbohydrates found in beer and wine. Further studies are warranted to explore the underlying mechanisms.

### Limitations and strengths

Some limitations should be noted in our study. First, the present study used a cross-sectional study design, and all data were self-reported. Therefore, we cannot rule out social desirability, interviewer, and/or recall bias. Also, according to previous literature [47, 48], there is a different susceptibility to famine mortality between adolescent males and females, and this difference could be another source of bias, which is not addressed in this cross-sectional study. Moreover, females exposed to stress like famine are more likely to have altered age of menarche commencement, shorted period of fertility, and an earlier onset of menopause. It may be worthwhile to consider these features in a follow-up longitudinal analysis, even though they are beyond the scope of this study. In addition, we did not include information about daily diets and birth weight in the model (because the CHARLS did not provide those relevant questions), which may potentially confound our findings. Thus, further studies with more comprehensive data are needed to validate the results. Lastly, we did not separate the participants by the severity of famine because we could not track the places they lived during 1959–1961. Despite these limitations, the strength of this study is the generalizability. This is a multy site study covering a large sample size which is representative of the entire country of China. Such significant findings extend our knowledge of the effect of famine exposure during adolescence on MetS and provide evidence on sex-specific interventions that may be used to prevent MetS among elderly Chinese.

## Conclusions

This study compared famine exposure to later MetS development among adolescent-exposed and non- adolescent-exposed groups. Famine exposure during adolescence was associated with a higher odds of MetS development in adulthood, but this association was only pronounced among Chinese males. The nutrition status in early life, including adolescence, may have a long-term effect and be associated with adverse health events in middle-to-late life. Targeting those eldely people who suffered famine during adolescence may help prevent the development of MetS in their later life. Future studies (e.g., animal experiments, cohort and longitudinal studies) are needed to further explore the underlying mechanisms.

## Data Availability

The datasets generated and analysed during the current study are accessible to researchers around the world on request in the CHARLS repository, [http://charls.pku.edu.cn/pages/data/2011-charls-wave1/en.html]. All data collected in CHARLS are maintained at the China Center for Economic Research (CCER), part of the National School of Development of Peking University, Beijing, China. Further information can be obtained with email charls_info@pku.edu.cn.

## Abbreviations

BMI: Body Mass Index
CHARLS: China Health and Retirement Longitudinal Study
CVD: Cardiovascular Disease
MetS: Metabolic syndrome
T2DM: Type 2 Diabetes Mellitus
